# Dispel the Clouds and See the Sun: Influencing Factors and Multiple Paths of User Retention Intention Formation

**DOI:** 10.3390/bs13100872

**Published:** 2023-10-23

**Authors:** Hongjin Zhang, Longying Hu, Yeom Kim

**Affiliations:** 1 School of Economics and Management, Harbin Institute of Technology, Harbin 150001, China; huly@hit.edu.cn; 2 School of Economics and Management, Harbin University of Science and Technology, Harbin 150001, China; yeom8909@163.com

**Keywords:** quality of experience, brand trust, brand attachment, user retention intention, case study, fsQCA

## Abstract

To achieve user retention through multifactor synergy, Internet enterprises must reduce costs and increase efficiency and sustainable development. In response to the dilemma that Internet companies are experiencing increasingly high user acquisition costs and serious user churn, this paper investigates a sample of 46,695 user reviews of nine product series from Xiaomi Ecological Chain. Case studies and qualitative comparative analysis are used to explore the influence mechanisms of quality of experience, brand trust, and brand attachment on users’ retention intentions. Our findings are as follows. (1) Brand attachment alone is not necessary for high user retention intention, but user perception, cognition, and brand trust are necessary. (2) Quality of experience positively impacts brand trust, attachment, and user retention intention. Therefore, improving user perception and cognition is critical in generating high user retention intention. (3) Five configuration paths can achieve high user retention intention, while three configuration paths lead to low user retention intention, and there is an asymmetric relationship between these paths. Among them, the role of quality of experience-driven configuration paths in generating user retention intention is the most prominent. (4) User perception and cognition can substitute with brand trust and attachment in the substitution relationship between configuration paths. Our findings have important theoretical and practical implications for revealing the realization paths of high user retention intention in Internet companies and provide a new perspective for future research.

## 1. Introduction

With the rapid advancement of mobile Internet and big data, users’ choices and access to homogeneous products and services have been significantly enriched. The conventional operational mindset has gradually rendered users “immune”, and the user flow dividend has gradually disappeared. User growth has shifted from focusing on “flow” to emphasizing “retention”, with the user retention rate emerging as a pivotal metric for measuring user growth, especially the retention behavior of loyal users, which is a critical reference for traditional platforms entering transformation and upgrade. From the user management perspective, having a loyal consumer, retaining users, and creating a compounding effect is key to every company’s growth and competitive advantage [1,2]. Furthermore, a high user retention rate not only reduces marketing costs [3] but also positively impacts word-of-mouth [4] and profitability [5]. However, what conditions lead to the formation of user retention intentions? Undoubtedly, this is an essential question for the business and academic communities to ponder.

The study of user retention originated in the 1980s when Fornell and Wernerfelt [1] were the first to explore the relationships between customer retention, satisfaction, and revenue, and this field subsequently attracted the attention of many scholars. Many studies have attempted to understand the meaning of user retention from the perspectives of time and retention ratio; researchers have argued that user retention is the behavior of starting to use an application at a particular time and continuing to use the application after a certain period [6], as reflected by the number of initial users who remain active for a given time [7]. Some scholars have also attempted to identify user retention mechanisms via survey methods. Previous studies have mainly used questionnaires or in-home surveys to obtain information about long-term retention [8], and modern companies commonly analyze user behavior data to develop user retention strategies [9]. In addition, studies have found that user satisfaction and loyalty are crucial for user retention [8], and the retention behavior of loyal users has a positive effect on traditional platforms for transformation and upgrading [10]. However, user satisfaction studies have reported controversial findings; some argue that satisfaction is the primary determinant of customer retention [11,12], but others propose a different or even the opposite view, arguing that satisfaction does not always positively affect customer retention [13] and that the relationship between the two is relatively weak [14].

Notably, numerous scholars have attempted to use empirical research methods to investigate the role of factors such as quality of experience, trust, attitude, and emotional attachment on user retention [15,16,17,18]. Studies have found that quality of experience (beyond perception) can resolve the customer satisfaction paradox from an affective perspective [19], and that quality of experience is the basis for retention [17]. Jang and Bae et al. [20]. Developed measures of service-oriented experience (integrated servitization experience, customized servitization experience, relational servitization experience, and playful servitization experience) are based on a service-oriented logic. They found that the servitization experience enables the co-creation of value with customers by positively influencing value-in-use. It allows long-term customer retention as customers form behavioral attachments and active pursuit toward the brand. Xu and Gu [21] found that a well-designed brand experience drives brand loyalty and leads to brand trust and attachment, thus leading to loyalty behavior. However, experience is a dynamic process, and the connotation and measurement of quality of experience vary significantly across disciplines, industries, and contexts [19], and form such factors as tourism experience quality [22,23,24,25,26,27], IoT experience quality [19,28], online shopping experience quality [29], library mobile user experience quality [30] and other research directions. Some scholars believe that screen content quality assessment is crucial in ensuring and improving the quality of experience, as perceived by end users. The rapid development of network and transmission technologies has facilitated a variety of multimedia applications and broadcast services and led to end-users expecting better quality of experience (QoE) from service providers. A consensus has yet to form on the constitutive dimensions of quality of experience [31], and further discussion regarding the microscopic mechanisms underlying the role of experience quality and user retention thus remains necessary [32]. In addition, Pan and Huang [16] found through a study of customers of experiential products that brand attachment and trust exhibited a significant correlation with user behavior. Brand trust and attachment reinforced continued purchase behaviors when satisfaction was low. The influence of consumers’ emotional attachment on their behaviors and attitudes can effectively promote repeat purchases, reduce customer retention costs, and enable companies to achieve stable revenue growth. Of course, whether users’ multiple emotions toward a product are synergistic or substitutes for each other and which emotions significantly impact user behavior are still being determined [16]. It has also emerged that brand trust is critical to maintaining long-term relationships between consumers and brands [33] and that consumer trust in a brand enhances intentions to patronize, co-creation, and brand loyalty [34,35,36].

It follows that although studies have identified relationships between quality of experience, brand trust, brand attachment, and user retention directly or indirectly from a single factor, there is no consensus on the definitions, connotations, or constituent dimensions of quality of experience, brand trust, brand attachment, or user retention intention [31,37,38,39]. Moreover, user retention occurs throughout an individual’s interaction with the product or service [40]. This behavior results not from a single factor but from the interaction of multiple factors. Therefore, the relationships between quality of experience, brand trust, brand attachment, and user retention intention deserve further exploration.

Based on problems observed in practice and the theoretical gaps in existing studies, this paper combines case studies and qualitative comparative analysis methods. It uses a sample of 46,695 user reviews of nine Xiaomi Ecological Chain product lines. We investigate the following questions: What are the conceptual connotations and mechanisms of quality of experience, brand trust, brand attachment, and user retention intention? To what extent are quality of experience, brand trust, and attachment necessary to generate high user retention intention? How do different sets of conditions achieve high and low user retention intention? This paper’s contributions are as follows. First, the connotations and mechanisms of action of quality of experience, brand trust, brand attachment, and user retention intention are deconstructed and revealed. Second, the configuration perspective identifies eight combined paths for generating high and low user retention intention. Finally, the effects of user perception and cognition on brand trust and attachment and the alternative relationships are revealed. Existing studies have mainly used traditional empirical research methods to test the relationships directly or indirectly between single factors, such as satisfaction, quality of experience beyond perception and brand trust, brand attachment, and customer retention. Such studies not only yield some contradictory research conclusions, but also ignore the fact that user (customer) retention results from the interaction of multiple factors. These studies lack the use of integrated methods to explore and verify the formation of customer retention mechanisms. We adopt a hybrid approach, combining a case study and fuzzy set qualitative comparative analysis (fsQCA) to reveal the connotations and mechanisms linking user perception, user cognition, brand trust, brand attachment, and user retention intention. We identify multiple equivalent paths to achieve high and low brand trust, brand attachment, and user retention intention from a configuration perspective, which helps shift the research field from a unidimensional perspective to a multidimensional holistic perspective. This study is also a valuable supplement to existing studies. In addition, our findings provide references and insights for startups and incumbents to choose appropriate configuration paths to improve user retention at different endowment stages.

## 2. Literature Review

### 2.1. Quality of Experience

Quality of Experience (QoE), a key concept in consumer behavior research [41] that provides evidence and judgment standards against which companies measure the experience effect [19], was first introduced in 1995 by Otto and Ritchie. They argued that quality of experience is subjective, holistic, intrinsic, general, symbolic, emotional, and the result of a customer’s psychological assessment or emotional response. The quality of experience explored was at the level of subjective perception and focused on emotions. Subsequently, ISO 9241-210-2010 [42], which is based on a cognitive perspective, considers the quality of experience as the user having perceived impressions and responses to the product, system, or service used or expected to use [42] and has gained more recognition from scholars. Some scholars have argued from the interaction perspective that quality of experience refers to customers’ subjective evaluation of their unique emotions formed during their consumption activities and interaction with the service environment, service providers, other customers, customers’ peers, and other elements [43].

Further, expectation confirmation theory suggests that the degree to which the outcome of the experience of using the product or service meets an individual’s expectations affects the individual’s future behavior [44]. Several scholars have found that the quality of experience significantly influences users’ behavioral intentions [41,45]. For example, based on service-dominant logic, Jang et al. [20] found that service experiences, through co-creating value with customers, can form long-term customer retention via customers’ behavioral attachment and active pursuit of brands. Similarly, some scholars have found that positive experiences lead to emotional or cognitive attachment to a brand, leading to repeat purchase behavior [46].

Previous studies have used different perspectives to investigate the connotations of quality of experience and its essential role in customer retention. However, due to the subjectivity and context-dependent nature of user experience [47], the existing literature on the quality of experience needs to be improved [43]. Further, although some scholars have investigated the relationship between quality of experience and user retention intention, the research is still at an early stage, and further study of the connotations and mechanism of the relationship is still needed.

### 2.2. User Retention Intention

User retention is critical issue in service management and relationship marketing [48]. It has an important impact on generation compounding effects in companies, but there is not yet a universally accepted definition in the literature [49]. Early scholars tried to understand and explain user retention from the perspectives of retention rate, customer retention, and customer reservation. For example, Rust and Zahorik [50] argue that retention is the tendency of customers to stay with a brand over time [50] and is measured by the length of time customers continue to purchase products from the company [51]. Some scholars argue based on the customer relationship management (CRM) perspective that CRM is the process of attracting customers and developing and maintaining customer relationships [52] through improving product performance, enhancing customer service, increasing customer concessions value and customer satisfaction, and establishing long-term, stable relationships of mutual trust with customers. On the one hand, CRM attracts new customers, maintains old customers, and improves efficiency and competitive advantage [53]. On the other hand, it can foster customer continuity and loyalty [54]. Some scholars emphasize that the core of CRM lies in effectively managing customer data through analyzing the data of various interactions between the enterprise and the customer in the entire marketing and sales process to provide support for business decision-making, enhance customer retention capabilities, improve customer awareness, and maximize revenue generation [55]. Scholars from the capability perspective believe that CRM capability involves integrating and leveraging customer resources, predicting customer behavior to attract potential customers, and establishing and maintaining relationships with them to enhance overall enterprise performance [56]. Others argue that customer reservation combines behavior and attitude [57], significantly impacting firm profitability [5]. For example, Jones et al. [58] define customer reservation as the willingness to repurchase, whereas Gerpott et al. [59] define it as the maintenance of the transactional relationship established between the firm and the customer, including customer purchase behavior and the tendency to maintain attitudes toward the future. User retention was introduced as a critical indicator to judge product value in Internet businesses. Markey [6] defined it as the behavior of a user who starts using an application at a certain period and continues to use it after a certain period. In contrast, Lin et al. [7] considers user retention as the number of initial active users [7].

Existing studies mainly examine user retention intention using two dimensions: time, and retention ratio. However, the formation of user retention intention is complicated by various factors, such as satisfaction [60], habit [49], and experience [20]. Therefore, existing studies have difficulty explaining it thoroughly. Consequently, further investigation is needed into whether factors other than time and retention ratio contribute to the formation of user retention intention.

### 2.3. Brand Trust

Trust is a highly influential concept in branding [61]. Brand trust is vital issue in fields such as psychology and marketing [33,62]. The concept was first introduced by Howard and Sheth, who argued that brand trust positively influences consumer intentions. However, the existing literature is very limited in its research on the sources, definitions, constitutive dimensions, and measurement methods of brand trust, and there is not yet a consensus [37,63]. Garbarino and Johnson [64] argue that brand trust is formed by customers’ positive experiences with a company’s products and services over time. Chaudhuri and Holbrook [34] argue that brand trust refers to the intention of the average consumer to rely on a brand’s ability to perform a given function. Delgado-Ballester [33], on the other hand, argues that brand trust is the reliance, expectation, confidence, and consumer behavior that consumers show towards a brand when exposed to risk. Shao et al. define brand trust based on an interaction perspective as the security consumers feel towards a brand when interacting with it [65]. Other scholars have found that brand trust originates from consumers’ brand perception, brand experience, brand value perception, brand knowledge, consumption habits, and virtual community trust [66,67,68,69,70]. Consumer trust in a brand reduces the perceived risk of the brand when purchasing and increases consumer confidence in the brand and its products [70]. It has also been argued that brand trust involves the ability of the supplier (brand) to fulfill its promises and maintain consistency in product and service performance and that brand trust affects the supplier’s brand loyalty and commitment [35,71].

Studies have confirmed the relationships between experience, brand trust, consumer behavior, and brand loyalty. However, they are still limited, especially regarding brand trust and its impact on users’ retention intention.

### 2.4. Brand Attachment

The research on brand attachment originated in psychology [39]. Bowlby [72] studied mother–infant relationships and found that attachment is an emotional bond between a specific individual (infant) and a specific object (mother). Schultz et al. [73] subsequently introduced the concept of attachment into marketing when studying the relationship between consumers and brands. Most scholars consider brand attachment to be a unique emotional bond that consumers have with a specific brand [74,75], and building consumers’ emotional attachment to a brand is an essential topic in brand management [76]. For example, Thach and Olsen [77] argue that brand attachment is a persistent preference and a psychological convergence of consumers towards a specific brand. Moreover, Pan and Huang [16] found that brand attachment is a self-associated, intensely relational emotional attachment that consumers develop in their interactions with brands. Attachment is conducive to reinforcing user purchase behavior. Some scholars, such as Wang et al. [39], consider brand attachment a pluralistic concept, arguing that brand attachment refers to consumers’ cognitive and emotional power to associate their self-concept with a brand, including various elements such as brand cognition, strong incentives, emotion, trust, repurchase intention, quality beliefs, word-of-mouth, and negative information resistance. Park et al. [74] found that this association developed based on cognition and emotion has a crucial role in forming brand loyalty.

Thus, there is not yet a consensus in the academic community on the conceptual definition of brand attachment. On the one hand, some scholars such as Brakus and Schmitt have explored the role of brand attachment from an experiential perspective [46]. They found that positive experiences lead to emotional or cognitive attachment to a brand, manifesting in repeat purchases and habitual behavior. On the other hand, Li et al. [78] found that consumers’ relational attachment to a brand strongly affects brand experience and repurchase intention. Therefore, enhancing consumers’ brand experience is a meaningful way to increase loyalty.

Studies have confirmed causal relationships between experience, brand attachment, and user behavior. Brand attachment is influenced by factors including consumer experience, sense of communicative fairness, consumer attitudes toward the brand, consumer brand function associations, negative online rumors, social benefits, and perceived value, etc. [79,80,81,82,83,84,85]. However, it is still necessary to identify the role of brand attachment in the relationship between quality of experience and user retention intention. Further exploration of its connotations and mechanism of action is still needed.

## 3. Methodology

This study analyzes the causes and conditions of users’ retention intention by understanding the quality of experience, brand trust, and brand attachment. Since the formation of user retention intention is based on multiple concurrent causal relationships, it is difficult to reveal the complex relationships between variables using traditional statistical analysis methods. Instead, case studies can help researchers explore and uncover the theoretical logic behind complex phenomena by making multiple observations of those phenomena that have not been fully explored in practice [86]. Meanwhile, the fsQCA method is more appropriate for addressing antecedent complexity and causal asymmetry [87]. It allows configuration analysis of similar cases with “multiple concurrent causations” to reveal possible combinations and interactions among influencing factors [88]. Therefore, this study uses a variety of case studies and qualitative comparative analysis and takes user retention intention as the explained unit to investigate its antecedents, including user perception, user cognition, brand trust, and brand attachment explanatory units.

### 3.1. Case Study

#### 3.1.1. Case Selection

This paper follows theoretical sampling principles and uses a competitive design [86,89]. For our case study, we selected the user reviews of Xiaomi Ecological Chain products for the following reasons. (1) Xiaomi has more than 500 million users, which represents a high user volume, indicating users’ recognition, acceptance, and trust of Xiaomi, and gives its user reviews high credibility. (2) Xiaomi has incubated more than 400 eco-chain companies, ranking first in the global consumer IoT market. Its business scope encompasses consumers clothing, food, housing, and transportation, so the research sample size is large, and its results are relatively universal. (3) The product development and release for Xiaomi and its eco-chain companies feature online and offline interaction with users, so we can trace each product’s development logic and user feedback, which provides a rich source of public data and strong availability of information. Therefore, Xiaomi is a highly suitable research object for this study.

We filter user comments according to the following principles. (1) The data mining object selects the product with the highest popularity [90]. (2) The selected user reviews are attribute-type comments based on objective facts about the product, provide more specific and clear attribute information, and analyze and explain specific properties [91]. (3) The selected user reviews are those written by senior evaluators with more experience, higher creditability, and higher review credibility than other evaluators [92]. (4) The selected user reviews are additional reviews with stronger perceived usefulness than the initial review [93]. (5) The selected user comments have received many likes or other user comments in response. (6) The selected user reviews include first-time purchasers, users who switched from other brands, and reviews posted by older users.

#### 3.1.2. Data Collection

User reviews, as the dominant factor in consumers’ online shopping decisions at this stage [92], are users’ expressions of their real feelings after purchasing products, services, or experiences, including information on product attributes, quality perceptions, cost performance, post-purchase experiences, emotional tendencies, and recommendation intentions. Compared with data obtained from other sources, user reviews have the advantages of comprehensive coverage, participants speaking voluntarily or anonymously, group thinking, and preservation [94]. Therefore, they can reveal the process of consumers’ feelings and self-construction. This study selects the user reviews of the highest-selling products in the Xiaomi Ecological Chain nine-product series as the data source and only the store with the highest sales volume is selected for each product. We use Python data mining tools to obtain 46,695 user reviews. Based on Park’s requirements for the relevance, understandability, adequacy, and objectivity of user review data, in [95], the authors organized the data for the 46,695 original user comments. The specific criteria include (1) deleting the duplicate comments of the same user according to the user’s name and the content of the comments. (2) Deletion of insubstantial content, such as irrelevant user comments. (3) Deletion of user comments whose descriptions are too unclear. (4) Deletion of untrue user comments. Finally, 671 valid data were obtained. Among these, the mined data originate from comments posted by star users with Plus membership level 1 and above in 2021–2022 to ensure the credibility of the data, as shown in Table 1.

#### 3.1.3. Data Analysis

To ensure the reliability of the research findings, this paper adopts the procedural theoretical approach proposed by Cobin and Strauss to discover and establish universal rules to explain the phenomena under study through objective, systematic observation and a standardized coding procedure [96]. This approach upholds a rational and open-minded attitude that can compensate for the lack of qualitative reliability and validity in the research [97]. Therefore, we completed two coding rounds to ensure the external validity of our data analysis. The first round of coding selected data from six product series (Xiaomi mobile phone, Redmi mobile phone, small household appliances, routers, and speakers, Xiaomi TV, and smart home devices) for open coding, axial coding, selective coding analysis, and user reviews of the Mi notebook, Xiaomi refrigerators, and washing machines, and Smart wearable devices kept for theoretical saturation tests. Furthermore, to eliminate personal subjective biases in the coding process, we used a three-person coding approach to code predetermined key variables independently. We then discussed the results and sought expert opinions to form the final coding results [98]. At the same time, to further enhance the theoretical and scientific nature of the results of this paper, the new concepts, categories, and relationships formed during the coding of cases were repeatedly compared with existing theories and literature to ensure the adequacy and robustness of the coding results. We then used a clear-set qualitative comparative analysis to assign values to the variables according to the dichotomous attribution principle. We then discussed the configuration path of antecedent conditions on user retention intentions through necessity analysis, adequacy analysis, and robustness tests.

Open coding compares, combines, and summarizes case material such that it can be conceptualized and categorized [98], as shown in Table 2. First, we numbered the user reviews according to product categories, with the following numbering rules: product category (1: Xiaomi phone, 2: Redmi phone, etc.), and user review serial number (e.g., 1-1 refers to the first user review of Xiaomi phone. Then, in the process of labeling, we followed the “original rules” to retain as much of the “original flavor “of the case material as possible and to preliminarily summarize it [97]. Next, we compared and refined the fragmented labeled codes in the conceptualization process and thus generalized the initial concepts. Finally, in the categorization process, the initial concepts with the same essential properties were clustered and named according to the concepts in the existing literature. This process results in 17 initial concepts and eight subcategories.

Axial coding analogizes the categorized concepts formed by open coding and establishes interrelationships between subcategories [97]. The two authors analyzed the above eight subcategories, explored their intrinsic connections at the conceptual level, categorized the codes through dialogue with the literature, and compared the coding results with any disagreements the coding team members discussed. This study further categorized the above subcategories into four main categories: Quality of Experience, Brand trust, Brand Attachment, and User Retention Intention, as shown in Table 3.

Selective coding is a further systematic treatment of the links between the categories that draws connections between the main types and distills a storyline that describes the phenomenon [97]. In this phase, the coding team undertook an in-depth analysis of the eight subcategories and four main categories mentioned above to understand the structural relationships between the main categories based on comparative interactions with the case material, as shown in Table 4.

**Figure 1:**
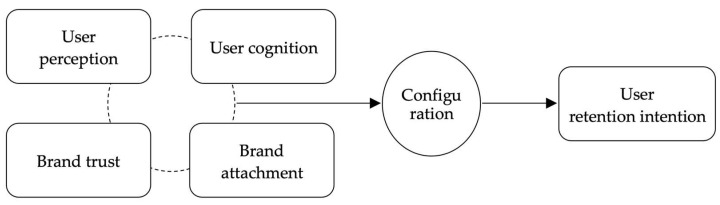
Analysis framework of the role of quality of experience and user retention intention.

Based on this, a clear storyline around the core category can be obtained (as shown in Figure 1): the user’s perception of product or service demand satisfaction, response efficiency, participation, and immersion depth. These factors, together with the user’s existing knowledge, preferences and expectations of the product or service, form the quality of the user experience. The quality of experience promotes user cognition of the product quality and product creators, which leads to trust in the brand (i.e., brand trust). At the same time, the quality of experience causes users to form an emotional attachment to and habitual consumption of the product or service (i.e., brand attachment). Users’ trust and attachment to the brand are further transformed into continuous attention and the tendency to purchase again (i.e., user’s retention intention).

#### 3.1.4. Theoretical Saturation Test

In this paper, to test the theoretical saturation of the model, we coded and iterated 224 user reviews for three major product lines: Xiaomi Notebook, Xiaomi Ice Wash, and Smart Wear. However, we did not find new categories or logical relationships frequently emerging. Therefore, the proposed theoretical model has reached saturation.

### 3.2. fsQCA Analysis

#### 3.2.1. Introduction and Applicability Analysis of fsQCA

Qualitative Comparative Analysis (QCA) is a method that integrates the advantages of quantitative and qualitative analysis methods based on set theory and Boolean algebra operations. It offers a systematic perspective for comparing multiple cases, providing theoretical explanations and methodological support for studying a phenomenon or outcome’s causative and antecedent elements. This research method aligns better with the nature of social phenomena. The key differences from other analytical methods are as follows: Firstly, it breaks through the traditional statistical methods that only examine the net effect of a single element or multiple elements on the outcome variable. Instead, it can analyze the multiple concurrent causal relationships between multiple antecedent variables and the outcome variable. Secondly, through necessity and sufficiency analyses, it overcomes the limitations of symmetrical thinking in conventional quantitative research by revealing the complex asymmetric relationship between antecedent variables and outcome variables. This approach also provides multiple equivalent path explanations for the emergence of the same outcome. Third, the method further validates the research hypotheses constructed based on the theoretical model and its test results, thereby cross-validating the correctness of the relevant model and hypothesis testing [106]. Compared with clear set qualitative comparative analysis (csQCA) and multivalued set qualitative comparative analysis (mvQCA), fsQCA further enhances the ability to analyze fixed distance and fixed ratio variables [87]. fsQCA can transform fuzzy set data into truth tables with almost no information loss, which allows truth tables to transform qualitative data for processing, simplify conditional configurations, and analyze finite diversity, giving the approach the advantage that it has properties of both qualitative and quantitative analysis [106]. The steps include identifying antecedent and outcome variables, data calibration, single antecedent variable necessity, adequacy analysis, conformational analysis, and interpreting the results.

This paper uses the fsQCA method to explore the influence mechanisms of user perception, user cognition, brand trust, and brand attachment on user retention intention, mainly based on the following reasons.

In contrast to traditional regression analysis methods, which emphasize the net effect of a single variable on the outcome and the existence of correlations among independent variables and can mask the unique effect of a single variable, the fsQCA method analyzes the causal relationship between conditional configurations and outcomes based on a holistic and systematic approach. In contrast, user retention intention forms throughout the interaction between users and the product or service [40]. Therefore, it results from a combination of factors, which falls within the scope of the question, “What are the conditional groupings that lead to the desired outcome?” Therefore, the choice of the fsQCA approach fits with the question addressed in this study [107].

The path of user retention intention formation is often not unique. Users can generate different choices in the process of interacting with products or services depending on their context. fsQCA methods, as a complement to traditional symmetry methods, can accommodate data asymmetry and identify potential interdependencies between antecedent conditions, thus revealing multiple equivalent symmetries to achieve the same outcome under specific conditions, which is consistent with the research questions in this paper [108].

Although traditional qualitative studies, such as single-case and multi-case studies, can analyze the research object in detail and depth, the research results are more specific, thus making it challenging to guarantee generalizability. In contrast, quantitative studies such as regression analysis have high reliability but relatively low validity and explanatory power of the phenomenon [108]. The fsQCA method can effectively combine qualitative and quantitative analysis techniques and obtain the configuration path that achieves high user retention intention through comparative analysis at the case level while ensuring that a sufficiently large sample size improves the reliability and validity of the findings in this paper.

#### 3.2.2. Definition and Calibration of Causal Conditions

Clear set qualitative comparative analysis, a comprehensive research strategy that combines the strengths of both qualitative and quantitative methods [106], explores how combinations of antecedent conditions lead to observable changes in the explained outcomes [88]. We discuss user perception, cognition, brand trust, and brand attachment configuration paths to achieve user retention intention. We followed the ‘dichotomous attribution principle’ [109] and referring to Mas-Tur and Pinazo’s [110] calibration of variables [106], we classified the antecedent conditions and outcomes in the 671 user comments as either 0 or 1. The value of 1 was assigned when the condition is satisfied or present, and 0 when the condition is not satisfied or current. This forms a definition and calibration of causal conditions table for the variables, as shown in Table 5.

#### 3.2.3. Necessity Analysis

To test whether antecedent conditions constitute necessary conditions for achieving a particular outcome, this paper analyzes the necessity of individual conditions (including their non-sets). A condition constitutes a necessary condition when the necessity of a single condition exceeds 0.9. The results of the analysis of the necessity of individual antecedent conditions influencing high user retention intention in Table 6 show that the consistency of user perception, user cognition, and brand trust are 0.98, 0.93, and 0.93, respectively, all of which exceed 0.9, indicating that these three antecedent conditions are necessary to explain high user retention intention. In analyzing the necessity of individual antecedents for influencing low user retention intention, the consistency of ~user cognition and brand attachment are 0.98 and 0.93, respectively, exceeding 0.9, thus suggesting that the above two antecedents are necessary to explain low user retention intention. The necessity of other individual antecedents for influencing high retention intention does not exceed 0.9, implying that these antecedents are unnecessary and that the concurrent synergistic effect of multiple conditions should be considered to indicate whether users will have retention intentions.

#### 3.2.4. Sufficiency Analysis

The sufficiency analysis of the configuration reveals the possible paths that lead to the multiple factors that constitute the results [87]. In this paper, we set the consistency threshold to 0.8. The frequency threshold is set to 1 [111]. This is based on the requirement of Scheider and Ragin to set the consistency threshold to no less than 0.75 and referring to the suggestion of Ragin to set the consistency threshold to greater than or equal to 0.8 [111], while meeting a PRI consistency of no less than 0.75 [87]. We used fsQCA 3.0 software to analyze the conditional configuration leading to high and low user retention intentions, as shown in Table 7. At the same time, we named the discovered configurations based on the configuration theorizing process [112].

(1) Longitudinal analysis of configuration paths generating high user retention intention

Table 7 presents the five configuration paths (S1a, S1b, S2, S3, S4) used to explain high user retention intention, where S1a and S1b constitute second-order equivalent configurations, i.e., they have the same core condition [88]. The overall consistency of the model solutions is 1, indicating that all five configurations are sufficient conditions for high user retention intention. Also, the comprehensive coverage of the model solution is 1, meaning that the antecedent condition largely explains the high user retention intention. The following longitudinal analysis details the path of each configuration that generates high user retention intention.

Quality of experience-driven. The configuration S1a (user perception*user cognition) indicates that a configuration path with high user cognition as the core condition and complementary high user perception as the second condition can generate high user retention intention, with a consistency of 1 and a coverage rate of 0.92, indicating that this configuration path has sufficient explanatory power for generating high user retention intention. When users have sufficient rational knowledge of the product or service, they will generate high retention intention if the product or service brings them a satisfactory perceptual experience. In this case, the user’s trust in and attachment to the brand is unnecessary to achieving a high retention rate. Typical users in this condition group are first-time buyers or users of a particular brand’s products and services or budget users who have switched from other brands and have a specific perception and value proposition of the target brand, product, and service.

Brand trust-assisted and user cognition-driven. Configuration S1b (user perception*brand trust) indicates that a configuration path with high user perception as the core condition and high brand trust as an assisting condition can generate high user retention intention. The consistency is 1, and the coverage rate is 0.88, indicating that this configuration path has sufficient explanatory power to create high user retention. This means that when users have sufficient rational cognition about the product or service and trust in product quality or the brand, they will have a high retention intention. In this case, the product or service does not need to bring users a satisfactory perceptual experience or for users to have an attachment to the brand to generate high user retention. Typical users in this condition configuration have a value proposition and personal preference for the target products and services. They have strong trust and emotional trust in the products, services, and even the brand founder of the specific brand.

User perception-driven and brand trust-driven. Configuration S2 (user perception*brand trust) indicates that a configuration path with high user perception and high user cognition as core conditions can generate high user retention intention. Its consistency is 1, and its coverage is 0.91, indicating that this configuration path has sufficient explanatory power for generating high user retention intention. This means that when users trust the brand’s product quality or the brand founder and when the product or service brings users a satisfactory perceptual experience, users will have a high retention intention. In this scenario, it is unnecessary for users to have rational perceptions of the product or service or to be attached to the brand to generate high user retention. Typical users in this condition configuration focus on the ultimate perceptual experience of the product or service and have formed a strong trust with the specific brand.

User perception-aided and brand attachment-driven. Configuration S3 (user perception*brand attachment) indicates that a configuration path with high brand attachment as the core condition and complementary high user perception as an assisting condition can generate high user retention intention. The consistency is 1, and the coverage is 0.66, indicating that this configuration path is a sufficient condition to create high user retention intention, which explains, to a large extent, the reason for its generation. When users express high emotional or cognitive attachment to a brand, they will have a high retention intention if the product or service brings them a satisfactory perceptual experience. In this scenario, it is unnecessary for the user to perceive the product or service rationally or to identify with the product quality or the brand founder. Typical users in this condition configuration are habitual users or fan users who have an emotional attachment to the products or services of a specific brand. They will continue to support the brand if the products or services of the target brand can always bring them a satisfactory perceptual experience.

User cognition-driven and brand attachment-driven. Configuration S4 indicates that a configuration path with high user perception and brand attachment as the core conditions can generate high user retention intention. The consistency is 1, and the coverage rate is 0.64, indicating that this configuration path is a sufficient condition to generate high user retention intention, which explains, to a large extent, the reason for its generation. When users have enough rational cognition about the product or service, they will have a high retention intention if they have a high emotional or cognitive attachment to the brand. In this scenario, the product or service does not need to give the user a satisfactory perceptual experience or for the user to have trust in the product quality or brand founder. Typical users in this condition configuration are rational users who compare and weigh the target products based on their experience and perceptions and are attracted to specific brands.

(2) Longitudinal analysis of configuration paths generating low user retention intention

The paths leading to low user retention intention have three configurations (W1, W2, and W3). Their model solutions’ overall consistency and coverage are 1, indicating that each antecedent condition has sufficient explanatory power to form low user retention intentions. Configuration W1 shows that the user retention intention is low in a configuration path with deficient user perception and cognition. Secondly, configuration W2 shows that users’ retention intention is low in a configuration path with deficient user perception, brand trust, and brand attachment. Finally, configuration W3 shows that users do not have a high retention intention in a configuration path with deficient user perception, brand trust, and brand attachment. This paper finds that the configurations W1, W2, and W3 show that deficiencies in user perception and cognition lead to low user retention intention, regardless of brand trust and attachment.

(3) Horizontal analysis of potential substitution relationships between conditions

By comparing the similarities and differences between the conditional configurations S1a, S1b, S2, S3, and S4, we can further identify the potential substitution relationships for the antecedent conditions, as shown in Table 8. The analysis shows that user perception and brand trust substitute for each other for products or services that provide users with good rational perceptions. For products or services that provide users with the ultimate perceptual experience, user perception, brand trust, and brand attachment substitute for each other. For products or services with sufficient rational perception, user perception, brand trust, and brand attachment again substitute for each other. User perceptions and cognition substitute for products or personal brands for which users express a strong trust. User perception and cognition substitute for brands for which users express high emotional or cognitive attachment.

The potential substitution relationships of user perception, user cognition, brand trust, and brand attachment conditions suggest that user perception and cognition have a more critical role. This is because both user perception and user cognition can play the same role as brand trust and attachment under the condition that users perceive and cognize the existence of a product or service, as shown in relationship categories two and four. In addition, the substitution relationship between user perception and user cognition revealed by relationship categories three and five further highlights user perception’s critical role and relevance in generating high user retention intention.

Finally, we investigate the “causal asymmetry” by analyzing the “non-set” (low user retention intention) of the explained variables and reveal that for products or services that bring poor perceptual experience to users, deficient user perception can substitute for the conditional combinations of deficient brand trust and deficient brand attachment. Likewise, for product or service users lacking sufficient rational cognition, user perception can be substituted with the conditional configurations of brand trust and attachment deficits. Finally, for brands lacking user trust, emotional and cognitive attachment, user perception and perception deficits substitute.

The potential substitution relationships among deficits of user perception, user cognition, brand trust, and brand attachment suggest that user perception and cognition deficits have critical roles. Both user perception deficit and user cognition deficit can play a role that only occurs when the combination of brand trust deficit and brand attachment deficit are also present under the condition of a lack of user perception and cognition of the product or service, as shown in the relationship categories six and seven. In addition, the substitution relationship between user perception deficit and user cognition deficit revealed in the relationship category eight further highlights the importance and relevance of user perception deficit in generating low user retention intention.

(4) Further analysis

Longitudinal analysis of configuration paths generating high brand trust. Table 9 presents the two configuration paths (S5 and S6) used to explain high brand trust, with overall consistency and coverage of model solutions of 0.94 and 0.93, respectively, indicating that the two configurations have sufficient explanatory power to form high brand trust. The following longitudinal analysis details each configuration path generating high brand trust.

User cognition-driven. Configuration S5 indicates that a configuration path with deficient user cognition and brand attachment as core conditions can generate a high brand trust. Its consistency is 0.98, and its coverage is 0.31. This configuration path indicates sufficiency for developing a high brand trust, partially explaining its generation. Users with sufficient rational cognition of the product or service will still generate a high brand trust, even if they lack a high emotional or cognitive attachment to the brand. In this case, the product or service does not need to give the user a satisfactory perceptual experience. Typical users in this condition configuration have value propositions and personal preferences for the target products and services. However, they may lack emotional attachment to the products or services of a particular brand.

Quality of experience-driven. Configuration S6 indicates that a configuration path with high user perception and cognition as the core conditions can generate high brand trust. The consistency is 0.94, and the coverage is 0.92, indicating that this configuration path has sufficient explanatory power for developing high brand trust. This means that when users have sufficient rational cognition about the product or service, if it also brings users a satisfactory perceptual experience, users will have high brand trust. In this case, users no not need an emotional or cognitive attachment to the brand. Typical users in this condition configuration focus on their ultimate perceptual experience of the product or service. They have value propositions and personal preferences for the target products and services.

Longitudinal analysis of configuration paths generating low brand trust. Configuration W4 shows that user perception, cognition, and brand attachment deficits are the configuration paths that generate low brand trust. Furthermore, the consistency and coverage of their model solutions are 0.85 and 0.35, respectively, indicating that this configuration is a sufficient condition for low brand trust and partially explains the causes of low brand trust.

Longitudinal analysis of configuration paths generating high brand attachment (Table 10). Configuration S7 demonstrates that high user perception, high user cognition, and absence of brand trust can develop high brand attachment. Its consistency is 0.91, and the coverage is 0.07, indicating that this configuration path is sufficient for generating high brand attachment, partially explaining its generation. When users have enough rational cognition about products or services, which gives them a satisfactory perceptual experience, they will generate high brand attachment even if they lack trust in the product or personal brand.

Longitudinal analysis of configuration paths generating low brand attachment. Configuration W5 shows that a configuration path of deficient user perception, and brand trust causes low brand attachment. The consistency and coverage of the model solutions are 0.97 and 0.11, respectively, indicating that this configuration is a sufficient condition for low brand attachment and partially explains the causes of low brand attachment.

#### 3.2.5. Robustness Test

Both changes in the consistency level and different calibration criteria affect the number of truth table rows (i.e., configurations) for logical minimization, which impacts the results. To ensure the scientific validity and robustness of the findings, the academic community has proposed testing the robustness of the conclusions by adjusting the calibration threshold, changing the consistency level, and using the Boolean minimization test [111]. This paper conducted robustness tests by increasing the case threshold, PRI consistency threshold, and Boolean minimization tests. The results can be considered robust if changing the consistency level or adjusting the calibration threshold results in a clear subset relationship between the configurations, even if the configurations look distinctly different and vice versa. At the same time, if the Boolean minimization robustness test does not show contradictory configurations, then the robustness test is passed.

**Figure 2:**
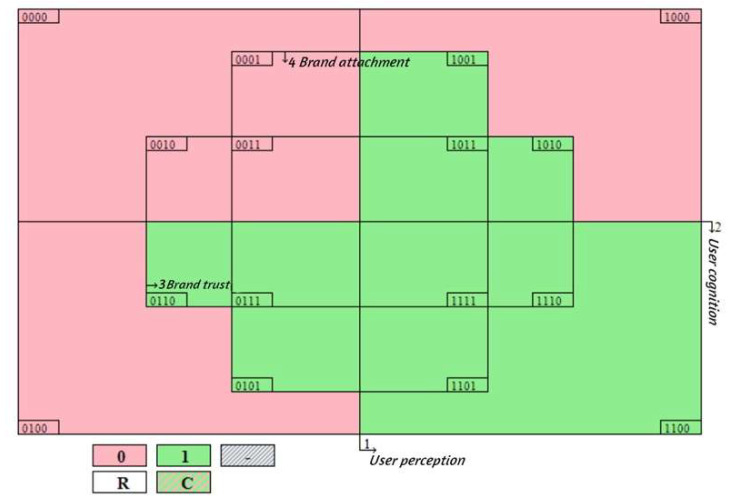
Venn diagram of the antecedent configuration of user retention intentions.

Firstly, the threshold of the number of cases is increased. In this paper, the threshold was increased from 1 to 2. The overall solution consistency level did not change, and the number of configuration paths changed from 8 to 7, as shown in Table 11. Although the individual configuration paths changed, the other seven configurations obtained after adjusting the case threshold were generally consistent with those before the adjustment. Secondly, the PRI consistency threshold is improved. After the PRI consistency was increased from 0.8 to 0.9, the overall solution consistency level did not change. The number of configuration paths and antecedent conditions was precisely the same, as shown in Table 12. Thirdly, the Boolean minimization test is performed. We performed a Boolean minimization test on the 671-sample dataset, and the truth table did not show contradictory configurations (i.e., for some observed cases, the result was [0]; in other practical cases, the result was [1]), as shown in Figure 2. In summary, the results of this study passed the robustness tests.

## 4. Conclusion and Discussion

### 4.1. Conclusion

This study’s research sample comprises 671 high-quality user reviews identified as having clear logic and strong persuasive power from nine series of Xiaomi Ecological Chain products. First, we explore the conceptual connotations and inner influence mechanisms of experience quality, brand trust, brand attachment, and user retention intention by using the programmatic grounded theory approach and then use a clear set qualitative comparative analysis, with user retention intention as the outcome variable, and user perception, user cognition, brand trust, and brand attachment as the explanatory variables. The necessity and sufficiency analysis identifies eight paths to achieving high or low user retention intentions based on user perception, brand trust, and brand attachment. The reliability of the study findings is also demonstrated by raising the case threshold, the PRI consistency threshold, and the robustness test of Boolean minimization. The main findings are as follows. (1) Quality of experience results from the interaction between user perception and cognition and plays a vital role in brand trust, attachment, and user retention intention. (2) Quality of experience positively impacts brand trust, attachment, and user retention intentions. However, improving user perception and cognition is more critical and general in generating high user retention intentions. (3) Five configuration paths can generate high user retention intention: quality of experience-driven; brand trust-aided and user cognition-driven; user perception-driven and brand trust-driven; user perception-aided and brand attachment-driven; and user cognition-driven and brand attachment-driven. Different configuration paths represent multiple ways of achieving high user retention intention.

### 4.2. Discussion

This study demonstrates, through case studies, that factors such as quality of experience, brand trust, and brand attachment significantly influence the formation of user retention intention. Moreover, it highlights that quality of experience can impact user retention intention by means of brand trust and brand attachment. These findings partially validate the existing literature’s proposition that quality of experience, brand trust, and brand attachment are key factors directly or indirectly affecting user retention (i.e., continued purchase behavior and patronage intention) [15,16,17,18]. However, previous studies have focused on the influence of specific experiences in specific scenarios on user loyalty behavior or sustained purchase behavior [46]. They have overlooked that experience quality results from user perception and cognition and that multiple elements contribute to forming user retention intention. This finding highlights the influence of experience quality on forming user retention intention at a more fine-grained level. Through configuration research, we discovered that apart from user perception, other factors may not be necessary for generating high/non-high levels of user retention intention, and in the substitution relationship of the configuration path, user perception and cognition can replace brand trust and attachment. Consequently, this suggests that users’ cognition regarding a product or service has a broader impact on whether user retention intentions are formed, and relying on a single-factor perspective to discuss the influence on user retention intentions is flawed. This paper argues that the formation of user retention intention mainly depends on the user’s perception level and cognitive ability towards the product or service. Furthermore, the stronger the user’s cognitive ability, the more it directly affects the formation of user retention intention.

In addition, this study proposes five configuration paths for achieving high user retention intention: experience quality-driven, user perception-driven with the assistance of brand trust, user perception and brand trust co-driven, brand attachment-driven with user perception, and user perception and brand attachment co-driven. The experience quality-driven path is identified as the core path for forming high user retention intention among these paths. This study demonstrates that the user retention intention formation process is diverse and encompasses five pathways. Furthermore, it emphasizes that improving the quality of user experience for products or services is the most effective path for achieving user retention intention. This conclusion provides strong evidence for addressing the customer satisfaction paradox [19]. However, it should be noted that the other four configuration paths are not directly explained in the existing literature. This paper argues that not all products or services offer users a high-quality experience. At this time, users’ trust and attachment to the brand will play a reinforcing role in the formation of users’ retention intention. This explanation validates previous research conducted by Pan and Huang [16], who discovered a significant correlation between brand attachment, trust, and users’ behaviors. Additionally, when users are dissatisfied with the product or service, brand trust and attachment can further strengthen their inclination to continue purchasing.

### 4.3. Theoretical and Management Implications

#### 4.3.1. Theoretical Implications

First, through a case study, this study finds that experiences quality is the result of the joint action of user perception and user cognition, thus expanding the experience quality literature from the single subjective level of experience perception to the subjective and objective level of the interaction between perception and cognition, which not only enriches the connotation and constitutive dimensions of the literature but also responds to Liu et al.’s [69] call to expanding experience quality research and provides a different theoretical perspective for related research.

Second, this study examines the role of multiple logical interactions between different dimensions of quality of experience with brand trust and attachment on forming user retention intention from a configuration perspective. This view is based on the theorization of histories [112]. It starts from the phenomenon itself and follows retrospective logic to investigate the histories of the causes of high user retention intention and whether individual antecedents constitute the necessary conditions for its formation. The four factors that affect user retention intention—user perception, user cognition, brand trust, and brand attachment—are incorporated into the same analytical framework, which lays a theoretical foundation for empirically exploring the relationship between multiple antecedents and user retention intention while providing an in-depth understanding of the combination of antecedents that generate high user retention intention.

Third, this paper responds to the call for a “combinatorial” methodology from the complex systems perspective [106,113] by introducing the histological perspective and the QCA approach to the study of user retention intention and combining them with a case study approach. This not only clarifies the equivalent histological paths and conditional substitution relationships that increase user retention intention but also allows us to explore the histological paths that lead to low user retention intention from a “causal asymmetry” perspective. It is also possible to explore the group state paths that lead to low user retention intention from the perspective of “causal asymmetry.” This further enriches the existing literature on the factors and underlying mechanisms that influence user retention intention using empirical research [19,48], providing new ideas and methodologies for research on issues related to the quality of experience and user retention.

#### 4.3.2. Managerial Implications

First, our research shows four concurrent synergistic effects on users’ perception and awareness of products or services, and trust and attachment to brands reveal the complexity of their retention intentions. This means that user management thinking should shift from “local optimization” to “configuration coordination” and focus on antecedent conditions by taking a holistic perspective according to the company’s business objectives and endowment. Therefore, we should focus on adapting the established antecedent conditions and form user retention strategies according to individual needs.

Second, this study proposes multiple paths to achieving user retention intention, which can provide a practical reference for start-ups and incumbents to choose the appropriate configuration path. Among the configurations, quality of experience plays a central role in forming brand trust, brand attachment, and user retention intention, and decision-makers must pay full attention to it. For start-up companies, it is difficult for users to form trust and attachment to the brand in a short period due to their relatively weak endowment in the early stage of their development, so we recommended creating product and service experiences for users that exceed expectations to enhance their retention intention. For incumbent companies, which have already formed a specific set of user resources and brand advantages, we recommended continuously iterating products and services with the best experience to enhance users’ trust and attachment to the brand while helping to reduce the loss of old users.

Third, brand attachment can increase users’ retention intentions, but focusing on this factor is unnecessary. Decision-makers need not pay too much attention to users’ attachment to brands but should focus on ensuring that users perceive and understand the value of products and services.

### 4.4. Limitations and Directions for Future Research

#### 4.4.1. Limitations

First, this study only explores the influence of four factors on user retention intention—user perception, user recognition, brand trust, and brand attachment—but user retention is a complex issue. The formation of user retention intention in different scenarios is not only related to users and brands; thus, but other influencing factors also need to be explored. Second, this study uses a combination of a case study and qualitative comparative analysis to reveal the internal construct process of user retention intention formation and thus lacks empirical research or other quantitative analysis to further validate the research findings. Finally, the sample was selected to study user reviews with the advantages of comprehensive coverage, participants’ anonymous speech, and groupthink. However, the sample size is still relatively limited, and there is a lack of data from cross-platform or multinational companies. Thus, different sample data may yield different results [114].

#### 4.4.2. Directions for Future Research

Future research can be considered from three aspects: The first is to enrich and improve the fundamental theoretical research on user retention in different scenarios. User experience quality, a new direction of user retention research that follows user-led logic, must be studied more deeply for different product types, user population attributes, consumption scenarios, operation strategies, intrinsic mechanisms, and evolution paths. Secondly, further combining user experience quality and behavior, especially in digital experience management, is an important entry point for the development of digital transformation of enterprises as well as determining how to improve the overall experience through monitoring and evaluating user experience quality and experience behavior. Third, acquiring and retaining new users is a hotspot in the current research. However, with the gradual disappearance of the traffic dividend, the cost and difficulty of acquiring new users are increasing. As such, maintaining the retention intention of old users and their potential value are worthy of further exploration.

## Figures and Tables

**Table 1:** Descriptive statistics of the selected products.

Category of Products	Product Number	Total User Reviews Mined	Number of Reviews After Data Cleaning	Number of First-Time Buyers	Number of Brand Switching Users	Number of Old Users	Average Number of Likes	Average Number of Comments	Additional Review Times
Xiaomi mobile phone	1	6930	143	9	28	106	21	11	11
Redmi mobile phone	2	7920	89	7	11	71	5	3	17
Small household appliances	3	4940	60	2	3	55	9	2	11
Routers and speakers	4	4850	83	5	3	75	6	2	24
Mi notebook	5	3960	41	4	6	31	11	7	9
Xiaomi refrigerators and washing machines	6	4226	31	2	5	24	2	1	19
Xiaomi TV	7	3960	91	6	5	80	2	13	5
Smart wearable devices	8	4950	66	4	4	58	6	2	11
Smart home devices	9	4950	67	1	4	62	6	2	8

**Table 2:** Open coding and theoretical sources.

Categorization	Conceptualization	Labeling	Original Information (Evidence Cited)	Literature Dialogue
User Perception	Demanding	Products to meet the diversified needs of users	[1–239] Superfine products, exquisite workmanship, touch-sensitive, fast running speed, powerful photo, and cost-effective, satisfying.	An and Li [99]
	Responsiveness	Companies respond quickly and efficiently to user requests	[4–2736] Xiaomi’s service attitude is excellent! I am a person who does not know anything about the network the first time to connect my own router. Customer service taught me step by step to do, only blame their IQ is too low, pounding for a long time did not understand the whole, people are very patient customer service repeatedly guide me, questions, and answers.	Parasuraman et al. [100]
	Participation	User involvement in product development research	[9–17] Nine years of meeting time, four years of companionship, from ordinary Mi fans to the inner test fans group, from the inner test fans group to the special group, met a lot of people along the way, which will be a lifetime hard to forget, not only to devote 100% of the feelings, or persistent persistence and love.	Shin [28]
User Cognition	User expectations	Product or service meets user expectations	[1–86] Xiaomi did not let me down. As good as ever. [4–1282] I bought many Xiaomi products, never disappointed, and will continue to support Xiaomi.	Hsu and Lin [101]
	Bounded rationality	Cost and benefit trade-offs the choice after balancing	[1–10] Rational consumption made me choose Xiaomi. The product itself is excellent. All aspects weighed to the extreme. The Xiaomi series are all good products worth choosing.	Fan and Lu [102]
Quality Trust	Reliable Quality	High quality wins user trust	[9–2189] I have always trusted Xiaomi’s products, the quality of workmanship is absolutely nothing to say, the future selection of small appliances preferred Xiaomi. [9–4402] I have always believed in the quality of Xiaomi. Every time I do not know how to choose will be more priority to choose Xiaomi.	Yuan et al. [103]
Competence Trust	Entrepreneurial competency trust	Users have confidence in the ability of entrepreneurs	[1–3381] Lei is my favorite entrepreneur. [2–6196] Support Lei as always. [1–2308] Lei Jun’s Xiaomi is still relatively generous. [2–2337] Lei Jun is a product of conscience.	Zhou et al. [104]; Yuan et al. [103]
Emotional Attachment	Emotional attachment	Users love the Xiaomi brand	[3–221] I have always had a soft spot for Xiaomi. [3–367] I have had a soft spot for the Xiaomi brand for years.	Park et al. [74]
Cognitive Attachment	Brand cognition	Positive brand experience makes users habitually consume	[9–337] My family has always been a Mi fan, from mobile phones to household appliances are Xiaomi. Anyway, Xiaomi is the best choice for shopping. [4–1484] Big brand, quality-assured, very satisfied, the future on Xiaomi.	Brakus and Schmitt [46]
User Retention Intentions	Retention commitment	Commitment to ongoing brand focus	[1–573] I will always support JD and Xiaomi. [4–2573] I always like Xiaomi’s stuff and will continue to support Xiaomi in the future.	This study refines
	Retention tendency	User propensity to repurchase after brand experience	[2–2397] I also use Xiaomi products. MIUI system is excellent, and I continue to buy Xiaomi and support the national product. [7–2260] Xiaomi is indeed a trustworthy product and cost-effective, and I will continue to pay attention to the brand Xiaomi and buy more Xiaomi products.	Rob [6]

**Table 3:** Axial coding and academic sources.

Main Category	Sub-Category	Relationship Connotation	Literature Dialogue
Quality of experience	User exception	User cognition and user perception reflect users’ cognitive impressions and experience results before and after using products or services, respectively, and the result of their joint action is the quality of user experience.	ISO 9241-210 [42]
	User cognition		
Brand trust	Quality Trust	Quality trust and ability trust reflect the user’s trust in the quality of the brand’s products and the ability of the brand builder after experiencing the products and services, respectively.	Yuan and Wang [103]
	Competence Trust		
Brand attachment	Emotional attachment	Emotional attachment and cognitive attachment reflect users’ positive emotional experience and cognitive impression of a specific brand, which manifests as loyalty and habitual consumption of the brand, constituting brand attachment.	Jiang et al. [105]
	Cognitive attachment		
User retention intention	Retention commitment	Retention commitment and tendency to stay reflect users’ commitment to stay tuned to the brand and the tendency to repurchase after a product or service experience, respectively, reflecting users’ retention intention.	Rust and Zahorik [50]
	Retention tendency		

**Table 4:** Examples of relationships between main categories.

Representative Original Statements	Main Category Relationship Structure
[4–1484] Big brand, quality-assured, easy to install, just three steps to get it done, the transfer speed is also quite fast. The price is a special discount to buy, excellent value. The mobile terminal can see to adjust many settings, and at a glance, very satisfied (user perception), later shop preferred Xiaomi brand (retention commitment).	User perception → User retention intention
[8–1121] I bought Xiaomi’s wearable products for the first time, and the experience was beyond my imagination (user expectations). I have also used Huawei and other brands of products before. In contrast, Xiaomi’s products are more cost-effective, more powerful, and practical (user perception), and the price is affordable. I also hope that Xiaomi’s brand is getting better and better and will continue to support the Xiaomi brand (retention commitment).	User cognition → User retention intention
[3–3323] Xiaomi’s products have consistently been recognized by my family and me as a very trustworthy brand, both in terms of quality of goods and after-sales service (brand identity). I will continue to buy Xiaomi’s products next time (tendency to stay).	Brand trust → User retention intention
[1–1843] Xiaomi mobile phone has always been my preferred brand (brand attachment). I have also been concerned about Xiaomi, used several Xiaomi mobile phones, and feel that the system has done very well. I will continue to support Xiaomi (retention commitment).	Brand attachment → User retention intention
[7–2260] TV received, the function is much, excellent, bought for the elderly and children to use, the language function is also very convenient and suitable, quickly get started, the picture quality is clear (user perception), a lot better than imagined (user expectations), Xiaomi is indeed a trustworthy product (brand identity), cost-effective (user perception), the future I will continue to pay attention to the brand Xiaomi (retention commitment), and buy more Xiaomi products in the future (retention tendency).	Quality of experience → Brand trust → User retention intention
[1–1463] The phone feels excellent in your hand. The shell is particularly delicate and silky smooth. The screen is particularly clear and worthy of Samsung’s screen. The software is high-speed (user perception). The camera is the best I have ever seen on a mobile phone (user perception). To sum up, this phone’s software and hardware are pretty good and very satisfactory. I am already a long-time Mi fan (brand attachment), and I will continue to patronize it in the future (retention commitment).	Quality of experience → Brand attachment → User retention intention

**Table 5:** Definition and calibration of causal conditions.

Variable Name	Definition	Judgment Notes	Assignment
User perception	The overall feeling of the user after experiencing the product or service.	The product or service gives the user a satisfactory perceptual experience.	1
		Poor perceived experience of the product or service to the user.	0
User cognition	Summary of users’ perceptions and experiences of tacit and explicit knowledge from review information [41].	Users express sufficient rational cognition about the product or service.	1
		Users express less cognition about the product or service.	0
Brand trust	Develops from past brand experiences and prior consumer-brand interactions [103].	Users express a strong sense of trust with the product or personal brand.	1
		Users do not express a strong trust with the product or personal brand.	0
Brand attachment	The strength of the cognitive and emotional ties that link consumers themselves to the brand [74].	Users express strong emotional or cognitive attachments to the brand.	1
		Users do not express strong emotional or cognitive attachments to the brand.	0
User retention intention	The tendency of customers to stay with the brand for a long time [51].	Users express a strong tendency to stay tuned or repurchase.	1
		Users do not express a strong tendency to stay tuned or repurchase.	0

**Table 6:** Analysis of the necessity of antecedent conditions for user retention intention.

	(High) User Retention Intention		(Low) User Retention Intention	
	Cons	Cov	Cons	Cov
User perception	0.98	0.99	0.12	0.01
~User perception	0.02	0.23	0.88	0.77
User cognition	0.93	0.99	0.02	0.00
~User cognition	0.07	0.50	0.98	0.50
Brand trust	0.93	0.99	0.17	0.01
~Brand trust	0.07	0.57	0.83	0.43
Brand attachment	0.67	0.99	0.07	0.01
~Brand attachment	0.33	0.84	0.93	0.16

**Table 7:** Configurations of antecedent conditions for user retention intention.

Frequency Cutoff: 1	(High) User Retention Intention					(Low) User Retention Intention		
	Consistency Cutoff: 1					Consistency Cutoff: 1		
	S1a	S1b	S2	S3	S4	W1	W2	W3
User perception	●		⚫	●		**⊗**		**⊗**
User cognition	⚫	⚫			⚫	**⊗**	**⊗**	
Brand trust		●	⚫				**⊗**	**⊗**
Brand attachment				⚫	⚫		**⊗**	**⊗**
Raw coverage	0.92	0.88	0.91	0.66	0.64	0.86	0.79	0.69
Unique coverage	0.004	0.01	0.03	0.01	0.003	0.19	0.12	0.02
Consistency	1	1	1	1	1	1	1	1
Overall solution consistency	1					1		
Overall solution coverage	1					1		

Note: “⚫” and “●” indicate that the condition exists, “**⊗**” and “⊗” indicate that the condition does not exist, blank indicates that the condition may exist or not exist, “⚫” and “**⊗**” represent the core conditions, “●” and “⊗” are auxiliary conditions.

**Table 8:** Potential substitution relationships between conditions.

Relationship Category	Configuration Comparison	Consistent Conditions	Potential Substitution Relationships between Conditions
1	S1a and S1b	User cognition	User perception ⇔ Brand trust
2	S1a, S2 and S3	User perception	User cognition ⇔ Brand trust ⇔ Brand attachment
3	S1a, S1b and S4	Brand trust	User perception ⇔ User cognition
4	S1b and S2	User cognition	User perception ⇔ Brand trust ⇔ Brand attachment
5	S3 and S4	Brand attachment	User perception ⇔ User cognition
6	W1 and W3	Lack of user perception	Lack of user cognition ⇔ Lack of brand trust + Lack of brand attachment
7	W1 and W2	Lack of user cognition	Lack of user perception ⇔ Lack of brand trust + Lack of brand attachment
8	W2 and W3	Lack of brand trust + Lack of brand attachment	Lack of user perception ⇔ Lack of user cognition

**Table 9:** Configurations of brand identity antecedents.

Frequency Cutoff: 3	(High) Brand Trust		(Low) Brand Trust
	Consistency Cutoff: 0.80		Consistency Cutoff: 0.85
	S5	S6	W4
User perception		⚫	**⊗**
User cognition	⚫	⚫	**⊗**
Brand attachment	**⊗**		**⊗**
Raw coverage	0.31	0.92	0.35
Unique coverage	0.01	0.61	0.35
Consistency	0.98	0.94	0.85
Overall solution consistency	0.94		0.85
Overall solution coverage	0.93		0.35

Note: “⚫” indicate that the condition exists, “**⊗**” indicate that the condition does not exist, blank indicates that the condition may exist or not exist, “⚫” and “**⊗**” represent the core conditions.

**Table 10:** Configurations of brand attachment antecedents.

Frequency Cutoff: 3	(High) Brand Attachment	(Low) Brand Attachment
	Consistency Cutoff: 0.91	Consistency Cutoff: 0.97
	S7	W5
User perception	⚫	**⊗**
User cognition	⚫	**⊗**
Brand trust	**⊗**	**⊗**
Raw coverage	0.07	0.11
Unique coverage	0.07	0.11
Consistency	0.91	0.97
Overall solution consistency	0.91	0.97
Overall solution coverage	0.07	0.11

Note: “⚫” indicate that the condition exists, “**⊗**” indicate that the condition does not exist, blank indicates that the condition may exist or not exist, “⚫” and “**⊗**” represent the core conditions.

**Table 11:** Robustness test (increasing the threshold of the number of cases from 1 to 2).

Frequency Cutoff: 2	(High) User Retention Intention					(Low) User Retention Intention	
	Consistency Cutoff: 1					Consistency Cutoff: 1	
	S9	S10	S11	S12	S13	W6	W7
User perception	●	⚫		⚫		**⊗**	
User cognition	⚫		●		●	**⊗**	**⊗**
Brand trust		⚫	●			**⊗**	**⊗**
Brand attachment				⚫	●		**⊗**
Raw coverage	0.92	0.91	0.88	0.66	0.64	0.17	0.79
Unique coverage	0.004	0.03	0.01	0.01	0.003	0.17	0.79
Consistency	1	1	1	1	1	1	1
Overall solution consistency	1					1	
Overall solution coverage	1					0.95	

Note: “⚫” and “●” indicate that the condition exists, “**⊗**” and “⊗” indicate that the condition does not exist, blank indicates that the condition may exist or not exist, “⚫” and “**⊗**” represent the core conditions, “●” and “⊗” are auxiliary conditions.

**Table 12:** Robustness test (improving the threshold of PRI consistency from 0.8 to 0.9).

Frequency Cutoff: 1	(High) User Retention Intention					(Low) User Retention Intention		
	Consistency Cutoff: 1					Consistency Cutoff: 1		
	S14	S15	S16	S17	S18	W8	W9	W10
User perception	⚫	⚫		⚫		**⊗**		**⊗**
User cognition	⚫		⚫		⚫	**⊗**	**⊗**	
Brand trust		⚫	⚫				**⊗**	**⊗**
Brand attachment				⚫	⚫		**⊗**	**⊗**
Raw coverage	0.92	0.91	0.88	0.66	0.64	0.86	0.79	0.69
Unique coverage	0.004	0.03	0.01	0.01	0.003	0.19	0.12	0.02
Consistency	1	1	1	1	1	1	1	1
Overall solution consistency	1					1		
Overall solution coverage	1					1		

Note: “⚫” indicate that the condition exists, “**⊗**” indicate that the condition does not exist, blank indicates that the condition may exist or not exist, “⚫” and “**⊗**” represent the core conditions.

## Data Availability

The data used in this study are derived from user review data publicly posted by the JD mall and has no privacy or ethical implications. The data presented in this study are available on request from the corresponding author.
